# A Low-Volume Epidural Blood Patch for the Treatment of Spontaneous Intracranial Hypotension: A Case Report

**DOI:** 10.7759/cureus.63059

**Published:** 2024-06-24

**Authors:** Jana Šimonová, Stanislava Jaselská, Róbert Šimon, Michaela Janková Šimonová

**Affiliations:** 1 1st Department of Anaesthesiology and Intensive Medicine, Faculty of Medicine, Pavol Jozef Šafárik University, Košice, SVK; 2 1st Department of Anaesthesiology and Intensive Medicine, Louis Pasteur University Hospital, Košice, SVK; 3 Department of Neurology, Louis Pasteur University Hospital, Košice, SVK; 4 1st Department of Surgery, Faculty of Medicine, Pavol Jozef Šafárik University, Košice, SVK; 5 1st Department of Surgery, Louis Pasteur University Hospital, Košice, SVK; 6 Department of Neurosurgery, Faculty of Medicine, Pavol Jozef Šafárik University, Košice, SVK; 7 Department of Neurosurgery, Louis Pasteur University Hospital, Košice, SVK

**Keywords:** orthostatic headache, epidural blood patch, conservative treatment, spontaneous intracranial hypotension, cerebrospinal fluid leak

## Abstract

Spontaneous intracranial hypotension (SIH) is a rare neurological syndrome. We report the case of a 47-year-old woman with acute, severe orthostatic headache after surgery, chemotherapy, and radiotherapy for breast cancer. The brain and spine magnetic resonance imaging showed signs of intracranial hypotension. We describe the results of a non-targeted epidural blood patch with 10 mL of the patient’s blood administered after unsuccessful conservative treatment. After the procedure, the patient reported gradual headache relief. This effect persisted over one year. The case shows that a single non-targeted low-volume epidural blood patch can be an effective treatment option for a patient with SIH when conservative treatment fails.

## Introduction

Spontaneous intracranial hypotension (SIH) is an increasingly recognized and highly disabling cause of headaches, with an estimated annual prevalence of 5 per 100,000 and a female predominance [1]. The peak incidence occurs around 40 years of age [2].

SIH is caused by cerebrospinal fluid (CSF) leak, but it is pathogenetically distinct from that of post-dural puncture headache or postoperative CSF loss [3]. There is a lack of understanding of its exact pathophysiology. The CSF loss is nearly always situated in the spine [1,4]. The main causes of CSF leaks include laterally located nerve root meningeal diverticula, ventral dural tears, or a direct connection between a nerve root sheath and the periradicular veins, called CSF-venous fistula [1,4].

Clinical features typically include postural headache, frequently occipital, frontal, or diffuse. Other symptoms include nausea or vomiting in 50.6%, neck pain or stiffness in 33%, tinnitus in 19%, and dizziness in 14%, along with hearing and visual disturbances, vertigo, back pain, and cognitive symptoms [5]. A typical clinical feature is a severe headache when upright, which is relieved when lying flat. Differential diagnoses of SIH include postural tachycardia syndrome, orthostatic hypotension, cervicogenic headache, and migraine [6].

At present, there is no single, absolutely reliable test or biomarker (blood or CSF test) that establishes the diagnosis of SIH [1]. Cheema et al. published a multidisciplinary consensus clinical guideline for best practice in the diagnosis, investigation, and management of SIH due to CSF leak [6]. Imaging modalities are necessary to determine this diagnosis. Magnetic resonance imaging (MRI) in patients with SIH is usually performed as a part of headache evaluation. The typical MRI signs of intracranial hypotension are S - subdural fluid collections, E - enhancement of pachymeninges, E - engorgement of venous structures, P - pituitary hyperemia, and S - sagging of the brain (SEEPS) [2,4,6,7]. In suspected cases of SIH, an MRI of the spine should be evaluated. If MRI is unavailable or contraindicated, computed tomography (CT) of the brain may show some of the findings supportive of the diagnosis and myelography could help locate the site of a spinal CSF leak [6]. It is useful for planning the therapy [7].

Conservative management for up to two weeks consists of bed rest, oral hydration, and caffeine intake of up to 900 mg a day. Non-steroidal anti-inflammatory drugs could also decrease the pain intensity. If an MRI of the spine identifies the site of the CSF leak, the next treatment options include a non-targeted or targeted epidural blood patch as soon as possible. Recent evidence suggests that the majority of patients with SIH respond to treatment with non-targeted epidural blood patches [6]. If this treatment does not provide long-term pain relief, a targeted application of blood or fibrin glue under X-ray or the surgical repair of the dural defect should be required.

## Case presentation

We present the case report of a 47-year-old woman with an invasive ductal carcinoma in her left breast. She underwent breast-sparing surgery in August 2021, chemotherapy until January 2022, and radiation therapy (whole-breast irradiation 5 × 5, 2 Gy and radiotherapy of the tumor bed 5, 2 Gy) until February 2022, currently being treated with exemestan. There was no trauma history. The patient did not undergo any surgical procedure except for breast surgery. There was a catamenial migraine in her anamnesis treated with nonsteroidal anti-inflammatory drugs (diclofenac or ibuprofen). She worked as a nurse.

Starting in January 2023, the patient reported a disabling postural bifrontal and occipital headache worsening with photophobia and nausea. At the beginning of her clinical problems, the patient was treated on an outpatient basis by a neurologist with the administration of infusion analgesic therapy consisting of ibuprofen 400 mg and paracetamol 1,000 mg intravenously daily, ibuprofen 400 mg tablets twice a day, and caffeine tablets 200 mg a day with no desirable clinical effect. She was referred for a brain CT scan with the conclusion of a suspected CSF drainage disorder. On January 31, 2023, after the brain CT scan, the patient was admitted to the Department of Neurology of Louis Pasteur University Hospital in Košice with a suspected diagnosis of SIH. Clinical examination on admission did not reveal any deficits.

**Figure 1:**
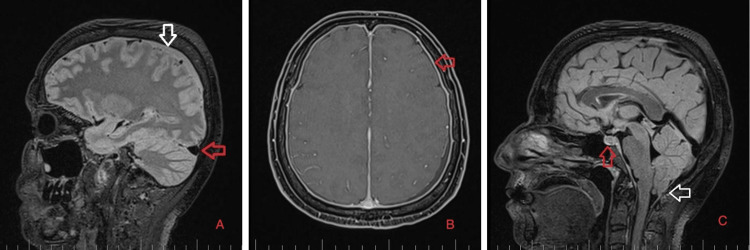
Brain MRI on February 1, 2023. A: Sagittal T2-weighted images. The white arrow shows subdural collection up to 5 mm in the parietal area, and the red arrow shows the distended convex appearance of the transverse sinus (signs of venous distension). B: Axial T1 images with contrast. The red arrow shows diffuse, smooth dural thickening and enhancement. C: Sagittal T2-weighted images. The red arrow shows slight inhomogeneity and pituitary gland enlargement, and the white arrow shows cerebellar tonsil herniation into the foramen magnum.

As part of the differential diagnosis, further imaging was performed. A brain MRI on February 1, 2023 (Figure 1) showed the following signs of SIH: subdural fluid collection up to 5.00 mm in the parietal area; diffuse, smooth dural thickening and pachymeningeal contrast enhancement; slightly homogeneous enlargement of the pituitary; cerebellar tonsillar descent into the foramen magnum; distension of the cerebral venous sinuses without thrombosis; and reduced fluid volume in the optic nerve sheath.

**Figure 2:**
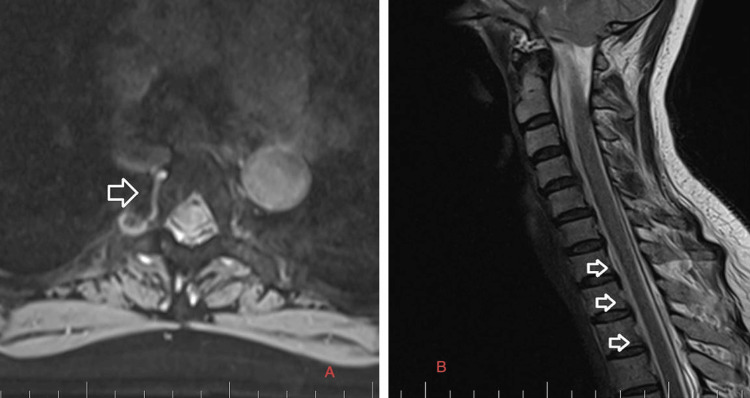
C spine MRI on February 2, 2023. A: Axial T2-weighted images. The white arrow shows the T1 right foraminal cerebrospinal fluid leak. B: Sagittal T2-weighted images. The white arrows show the spinal longitudinal extradural fluid collection type 1b.

Precisely locating the site of the CSF leakage is fundamental to successful treatment [8]. Hence, on February 2, 2023, we performed a C spine MRI. At the level of C7/T1/T2, a T1 right foraminal CSF leak, as well as the spinal longitudinal extradural fluid collection (SLEC) caudal to the C5 level was detected (Figure 2). This finding confirmed the CSF leak outside the dural sac. It was an SLEC 1b type of CSF leak [4].

Conservative treatment lasted 10 days and consisted of intravenous balanced crystalloid fluids 2,000-2,500 mL a day, caffeine tablets at a daily dose of 200 mg, bed rest regime, non-opioid drugs metamizole 3 g intravenously a day, ibuprofen 1,200 mg intravenously a day, and paracetamol/caffeine 500 mg/65 mg tablet. This treatment was unsuccessful. Therefore, on February 10, we applied a non-targeted lumbar epidural blood patch (EBP). The patient was lying in the left lateral decubitus position. Under strict aseptic conditions, the epidural space was detected by the loss of resistance technique with an 18 G Tuohy needle at L2/L3 level, and 10 mL of autologous venous blood was applied by an experienced anesthesiologist into the epidural space. The patient tolerated the procedure well. We left the patient lying in the supine Trendelenburg position for four hours. Heart rate, non-invasive blood pressure, and pulse oximetry were monitored during and after the procedure. The patient reported gradual improvement of the headache. On February 12, after a neurological assessment, the patient was discharged from the hospital.

**Figure 3:**
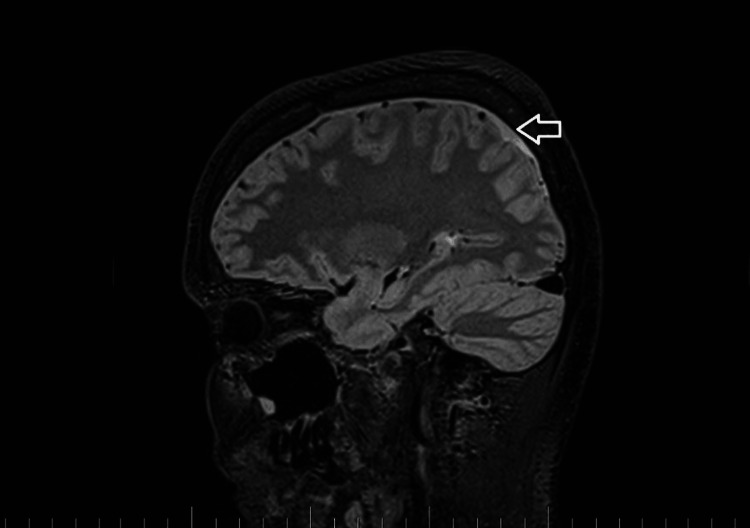
Brain MRI on March 5, 2023. Sagittal T2-weighted images. The white arrow shows a regression of the subdural fluid collection in the parietal area to 2.5 mm.

A brain and C spine MRI scan on March 5, 2023 (one month after the procedure) showed a regression of the subdural collection from 5.00 mm to 2.5 mm in the parietal area but all other parameters remained unchanged (Figure 3).

**Figure 4:**
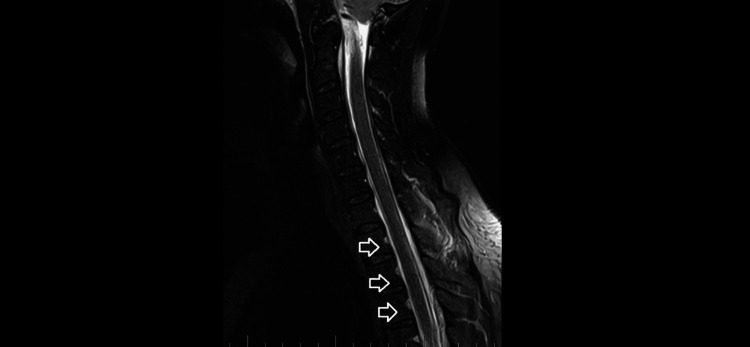
C spine MRI on March 5, 2023. Sagittal T2-weighted images. The white arrows show the regression of spinal longitudinal extradural fluid collection.

MRI myelography (Figure 4) showed no CSF leak. The SLEC was almost totally absorbed and only a tiny residuum persisted. The patient reported significant headache relief.

At six months follow-up, in September 2023, the patient was almost asymptomatic, taking only 100 mg of caffeine medication a day. Because of a dural rupture, she was seen by a rheumatologist, who ruled out general connective tissue disorders.

**Figure 5:**
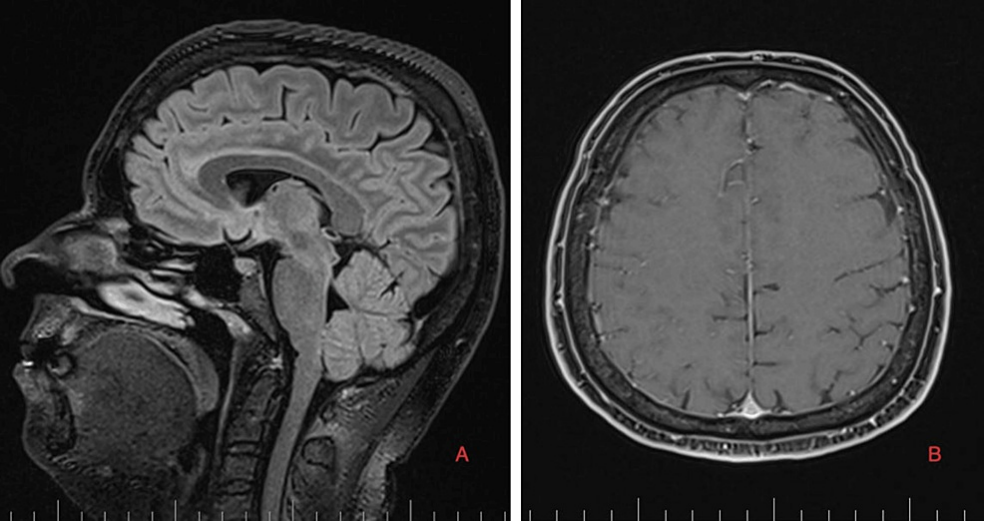
Brain MRI on September 4, 2023. A: Sagittal T1 with contrast images showing total regression of spontaneous intracranial hypotension signs. B: Axial T1 with contrast images without any pachymeningeal enhancement.

**Figure 6:**
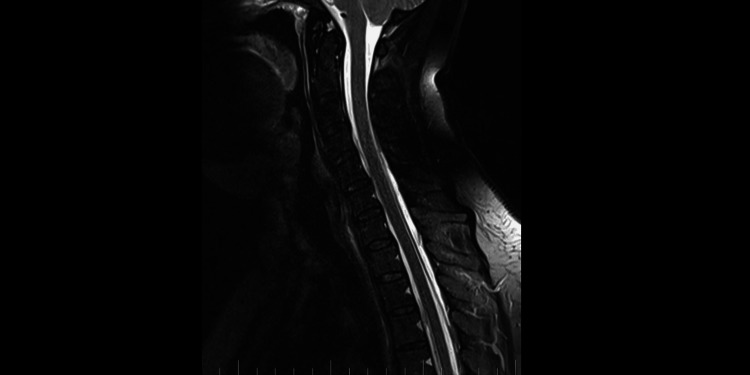
C spine MRI on September 4, 2023. Sagittal T2-weighted images showing total spinal longitudinal extradural fluid collection resorption.

Brain and C spine MRI (Figures 5, 6) on September 4, 2023, showed a total regression of SIH signs. MRI myelography showed complete resorption of the SLEC, with no CSF leak. The patient was without any clinical symptoms or headache. She continues to work as a nurse. Her quality of life has improved significantly.

## Discussion

Subdural collections are frequent MRI or CT findings but can be interpreted as primary rather than secondary pathological entities [9]. SIH is a rare syndrome with an annual incidence of approximately 5 per 100,000 and peaking around the fourth or fifth decade of life, although it can occur at any age. It is slightly more common in women. Although the true pathophysiology remains unclear, it is likely in most patients that a tear in the dura matter allows CFS leak and subsequent intradural CSF hypovolemia [9]. SIH usually occurs due to spontaneous CSF leaks typically located at the nerve root sleeves at the cervical or thoracic spine levels. It is thought to occur following trivial trauma, such as sporting activities, trivial falls, coughing, or sneezing, though the exact cause remains unknown [7]. The correlation between SIH and connective tissue disorders supports the hypothesis of a dural compliance disorder as the main cause of this syndrome [10].

In a review of clinical features, 8% of patients had a non-postural headache, and 3% did not experience a headache [5]. Headache generally occurs or worsens within 15 minutes of assuming the upright position and improves within 15-30 minutes after lying down [1]. Headache is frequently occipital, frontal, or diffuse. Many other presenting symptoms have been reported.

Numerous cranial MRI signs of SIH have been described. As almost all SIH cases are caused by spinal CSF leaks, spinal MRI is complementary to head scans. Some patients have spinal CSF leaks but do not show SIH signs in head scans; therefore, with appropriate clinical symptoms, both head and spinal MRI scans should be performed [2]. Dobrocky et al. described a novel scale based on MRI findings to help decide the need for invasive techniques to identify a possible CSF leak in patients presenting with postural headaches. They analyzed nine quantitative parameters and seven qualitative parameters. Enhancement of the pachymenginges, engorgement of venous sinus, and effacement of the suprasellar cistern of 4 mm or less, as major findings, were assigned a score of 2, while subdural fluid collection, effacement of the prepontine cistern of 5 mm or less, and mamillopontine distance of 6.50 mm or less were considered minor and assigned 1 point each. Patients with 5 points on a scale of 9 points or more have a high probability of having a CSF leak [11].

Our patient suffered from severe postural headaches, though she had no history of any trauma or fall, and a rheumatologist excluded connective tissue disorders. MRI showed numerous signs of SIH, and according to Dobrocky’s scale, a minimum of 5 points were found. Considering the confirmed leak of the SLEC 1b type and the absence of discopathic lesions or osteophytes in the location of the CSF leak, while taking into account the negative rheumatological examination, we assume that the radiation therapy could be the cause of dural insufficiency at the site of the nerve root sheath. The patient continues to be checked by a neurologist due to the risk of SIH recurrence.

A robust treatment paradigm for SIH is yet to be agreed upon [6]. In 15-30% of all SIH cases, conservative management lasting one to two weeks could be successful. This includes strict bed rest, plenty of caffeine intake, theophylline, analgesics, and sufficient intravenous or oral hydration [12,13].

EBP has been shown to be effective in the treatment of SIH for many years [14]. A non-targeted EBP should be offered to all patients with clinical or imaging diagnosis of SIH after no more than two weeks of conservative management [11,13]. Two possible mechanisms can explain the effectiveness of EBP. Immediate tamponade effect from the injected blood causes compression of the thecal space with a quick increase of lumbar and consequently intracranial CSF pressure. Long-lasting effect by the plugging of the dura opening with the creation of a blood clot caused by the interaction between the injected blood and procoagulant components in the leaking CSF [15]. Perthen et al. analyzed the treatment of 73 patients with SIH. Sixty-six underwent EBP, with 39 (59%) having a good response. The relapse rate was 10% [16].

No improvement of symptoms of SIH after a one-week trial of conservative management is an indication for performing EBP [7]. Perthen et al. recommended not delaying the non-targeted EBP once a firm diagnosis has been made [16].

The volume of blood depends on the site of placement and is approximately 4-6 mL at the cervical, 10-12 mL at the thoracic, and 20-30 mL at the lumbar region. Large EBP volumes of more than 20 mL provide higher success, but there is no difference between targeted and non-targeted EBPs [7]. Perthen et al. recommend performing a non-targeted EBP in the lumbar region by an obstetric anesthetist, with an aim to inject at least 30 mL of blood. If the first blood patch produces only transient improvement, a second blood patch is offered [16]. Cheema et al. applied up to 40 mL of blood, ideally at a minimum total volume of 20 mL. The administration of autologous blood should be ceased when the patients experience back pain or pressure, headaches, or radicular symptoms that they can no longer tolerate [6].

In our case report, non-targeted EBP was performed in the lumbar region. We successfully applied only 10 mL of blood. The decision to apply only 10 mL of blood during the first EBP was based on our experience with the treatment of post-dural puncture headaches in the obstetric population after an unintentional dural puncture during labor epidural analgesia. In these cases, this is a targeted EBP because we applied the EBP at the site of the CSF leak. Had the first EBP been ineffective, we planned to apply 20 mL of blood during the next application. Administering a lower volume of blood could be associated with a lower risk of neurological complications resulting from the formed epidural hematoma. However, EBP carries the potential risk of inducing rebound intracranial hypertension [17].

Optimal EBP volume is unknown and likely varies among patients due to patient factors such as age, degree of spine spondylotic changes, and relative size of the dural hole. There is a lack of correlation between EBP volume and success rate [18].

EBP must be considered as the basis in the treatment of SIH and a single EBP is effective in 64% of patients. Non-responders may require multiple EBPs. The number of EBPs before determining failure and the time gap between performances of subsequent EBP are unclear [7]. If there is no response or only a transient response to the first EBP, a second EBP could be considered before proceeding to myelography. The recommended interval between EBPs varies from 48-72 hours [5] to 2-4 weeks [6].

Perthen et al. stated that although treatment responses are assessed clinically rather than radiologically, some patients underwent repeated imaging after clinical improvement, sometimes for unrelated reasons, and they reported that normalization of the imaging appearances mirrors clinical response [16]. It is clear that the clinical effect of the treatment begins significantly earlier than the adjustment of the radiological signs of SIH, which requires at least a six-month interval from the administration of EBP. In our patient, significant headache relief occurred within two weeks, although radiological findings one month after the procedure improved, but persisted. We decided to wait and not repeat the EBP. After six months, the brain and spine MRI became normal.

## Conclusions

SIH is uncommon and requires a high index of suspicion for diagnosis. It can occur following trivial trauma, neck chiropractic manipulation, sporting activities, and coughing. Sometimes the exact mechanism is unknown. Connective tissue disorders and radiation therapy can also cause weakening of the dura mater. A typical symptom is a sudden onset of postural headache. Neurological examination is needed, and MRI of the brain and the spine are the main diagnostic tools. When the diagnosis is made and conservative treatment is ineffective, a non-targeted EBP is indicated. Although the appropriate volume of blood is unknown, 20-40 mL is most often used in the lumbar region. This case report points out that even a low-volume EBP can be effective and safe in the treatment of SIH.
